# Pathological observation and transcriptomic analysis of thymus injury in PRRSV-infected piglets

**DOI:** 10.1007/s11259-023-10133-x

**Published:** 2023-06-02

**Authors:** Naying Su, Zhengdan Lin, Xi Liu, Xiuxiu Sun, Xinxin Jin, Helong Feng, Cunlin Zhan, Xueying Hu, Changqin Gu, Wanpo Zhang, Guofu Cheng

**Affiliations:** 1 College of Animal Medicine, Huazhong Agricultural University, Wuhan, Hubei Province China; 2 Shanghai InnoStar Bio-tech Co., Ltd., Shanghai, China; 3 Hubei Academy of Agricultural Sciences, Wuhan, Hubei Province China

**Keywords:** Porcine reproductive and respiratory syndrome virus, Piglets, Thymus, Transcriptome, Histopathological findings

## Abstract

The thymus, the central immune organ in mammals, plays an important role in immune defense. Porcine reproductive and respiratory syndrome virus (PRRSV) infection in piglets can cause thymus injury and immunosuppression. However, the mechanisms of thymus injury remain unknown. This study was aimed at investigating the specific manifestations of thymus injury through the construction of a PRRSV-infected piglet model and histopathological observation. In this study, fourteen 40-day-old PRRSV-free piglets were randomly divided into two groups, eleven of which were intramuscularly injected with 3 mL of PRRSV WUH3 virus suspension (10^6^ PFU /mL) in the infection group, and three of which were sham-inoculated with 3 mL of RPMI-1640 medium in the control group. Clinical necropsy and samples collection were performed on day 8 after artificial infection. With the Illumina platform, the transcriptomes of piglet thymus tissues from infected and control piglets were sequenced to explore the relationships of differentially expressed genes (DEGs) and signaling pathways with thymus injury. The immune organs of PRRSV-infected piglets were severely damaged. The histopathological findings in the thymus indicated that PRRSV infection was associated with a large decrease in lymphocytes, cell necrosis and cell apoptosis; an increase in blood vessels and macrophages; thymic corpuscle hyperplasia; and interstitial widening of the thymic lobules. The transcriptomic analysis results revealed that the Gene Ontology functions of DEGs were enriched primarily in biological processes such as angiogenesis, regulation of angiogenesis and positive regulation of cell migration. Moreover, greater numbers of blood vessels and macrophages were observed in the thymus in PRRSV-infected than control piglets. KEGG pathway enrichment analysis revealed that the DEGs were significantly enriched in the Toll-like receptor signaling pathway, chemokine signaling pathway, IL-17 signaling pathway and TNF signaling pathway. The expression of TLR8, IRF5, the chemokines CCL2, CCL3L1 and CCL5; and their receptors CCR1, CCR2 and CCR5 was significantly up-regulated in PRRSV infection, thus suggesting that these cytokines were associated with the pathological processes of thymus injury.

## Introduction

Porcine reproductive and respiratory syndrome virus (PRRSV) is an enveloped, single-strand positive-sense RNA virus belonging to the family *Arteriviridae* and genus *Arterivirus*, which is divided into PRRSV1 (European genotype PRRSV) and PRRSV2 (North American genotype PRRSV) (Rossow 1998; Dokland 2010). PRRSV2 is the main genotype in China and has been circulating decades (Bao and Li 2021). PRRSV WUH3 was isolated from the brains of pigs in China in 2006, and identified as a highly pathogenic North American genotype PRRSV (Li et al. 2009). Porcine reproductive and respiratory syndrome (PRRS) is an infectious disease caused by PRRSV, which is characterized by reproductive disorders in sows, respiratory disease in piglets and immunosuppression (Shi et al. 2010; Zimmerman et al. 1997). The interaction between PRRSV nonstructural protein and host protein allows the virus to escape from the host immune system (Ma et al. 2021). PRRSV affects the host immune system, thereby resulting in decreased immunity or immunosuppression, and consequently secondary infection with bacteria, such as *Actinobacillus pleuropneumoniae*, *Haemophilus parasuis* or *Streptococcus suis*, and co-infection with other pathogens, such as pseudorabies virus, porcine circovirus 2, swine influenza virus or porcine microvirus in pigs (Zhao et al. 2021).

PRRSV first invades the alveolar macrophages of the host lung, then migrates to other organs and tissues through blood circulation and lymphatic circulation (Lunney et al. 2016). Beyond the lungs, immune organs are the main infection sites of PRRSV, and the virus can persist in immune organs (Xiao et al. 2004). The thymus, the central immune organ in mammals, and the site of T lymphocyte differentiation, development and maturation, plays an imperative role in immune defense (Luo et al. 2021; Matloubian et al. 2004). The development of the thymus differs among animal species. In pigs, the thymus gradually atrophies with age and eventually degenerates (Wang et al. 2019). Pathological atrophy of the thymus also occurs when animals are infected with viruses (such as bovine viral diarrhea virus), bacteria (such as *Salmonella typhimurium*), parasites (such as *Trypanosoma cruzi*) or fungi (such as *Aspergillus fumigatus*), thus leading to a weakened immune response to pathogens or vaccines (Luo et al. 2021). PRRSV can infect piglets through horizontal and vertical transmission, thereby damaging the thymus and preventing piglets from establishing a normal immune response (Vilalta et al. 2019; Ruedas-Torres et al. 2020).

PRRSV-induced apoptosis in the lymphoid organs, such as the thymus and lymph nodes, is the main cellular mechanism of immunosuppression (Ruedas-Torres et al. 2020). Pregnant sows infected with PRRRSV may lead to cell apoptosis in the heart, liver and thymus of the fetus (Malgarin et al. 2021). Multiple signaling pathways, such as Bcl-2 family protein-mediated mitochondrial pathway, PI3L/Akt pathway and TNFR1/Fas-mediated death receptor pathway, contribute to PRRSV-induced apoptosis (Fan 2019). The co-expression of immune checkpoints *PD1/ PDL1, CTLA4, TIM3, LAG3 and IDO1* in the thymus of the PRRSV-infected piglets affected T cell development and negatively regulated the host immune response aginst PRRSV (Ruedas-Torres et al. 2021).

The RNA sequencing (RNA-seq) technique is widely used in transcriptomic research because of its advantages of high-throughput and high sensitivity (Sudhagar et al. 2018). Transcriptomic analysis has been widely used in the study of the molecular mechanism of PRRSV. However, PRRSV-associated transcriptomic studies have often focused on lung or porcine alveolar macrophages (PAMs), whereas relevant transcriptomic analysis of the thymus has been less commonly reported (Cong et al. 2014; Li et al. 2015). PRRSV infection leads to reproductive disorders in sows, respiratory damage in piglets and immunosuppression. It seriously endangers the health of pigs and the economic development of pig industry. The thymus, an important central immune organ, is significant in resisting pathogen invasion and maintaining immune function. PRRSV infection cause thymus damage, which is a key reason for decreased immunity and secondary infection in pigs. However, the specific manifestations and exact mechanism underlying thymic injury has not been elucidated. In this study, we constructed a PRRSV-infected piglet model and determined the specific manifestations of thymus injury caused by PRRSV, on the basis of macroscopic examination, histological observations and immunohistochemical analysis. In addition, we performed transcriptomic analysis of the piglet thymus to investigate the associations of differentially expressed genes (DEGs) and signaling pathways with histopathological phenomena of thymus injury. This study contributed to understanding of the specific manifestations and the molecular mechanisms of thymus injury caused by PRRSV infection in piglets.

## Materials and methods

### Animals, virus and experimental design

Fourteen 40-day-old PRRSV-free piglets were obtained from a local farm in Hubei Province. All piglets were fed in the condition of consistent growth environment, feeding management and dietary nutrition levels. The piglets of the same genetic background and similar weight (15-17 kg) were selected for the experiment. These piglets were randomly divided into two groups, 11 of which were intramuscularly injected with 3 mL of PRRSV WUH3 virus (GenBank no. HM853673.2) suspension (10^6^ PFU /mL) in the infection group, and three of which were sham-inoculated with 3 mL of RPMI-1640 medium in the control group. The two groups were housed separately and given adequate feed and water. After the challenge, the body temperature of piglets was measured daily at 16:00, and clinical signs were observed and recorded.

### Animal autopsy and sample collection

Piglet weighing, anterior vena cava blood collection and clinical necropsy were performed on day 8 after artificial infection. The thymus, hilar lymph nodes, inguinal lymph nodes, mandibular lymph nodes, tonsils and spleen were weighed, and the lesions of each tissue were recorded. Samples were fixed in 4% paraformaldehyde buffer. Samples snap frozen in liquid nitrogen were subsequently transferred to −80 °C for storage.

### Histopathological observations

The samples were dehydrated, cleared, wax-infiltrated, embedded and serially sectioned into 3 μm paraffin sections. The lesions were observed and recorded through microscopic examination after hematoxylin-eosin (H&E) staining.

### Immunohistochemistry (IHC)

Paraffin sections were dewaxed and then thermally repaired in citrate buffer (pH 6.0) or EDTA buffer (pH 9.0). After washing with PBS, peroxidase blocker (Gene Tech, Shanghai, China) was added for 15 min. Blocking was performed with goat serum (Boster Biological Technology, Wuhan, China) for 30 min. After incubation with primary antibodies overnight at 4 °C, sections were incubated with secondary antibodies (Gene Tech, Shanghai, China) for 30 min at room temperature. The reaction was visualized with DAB chromogen (Gene Tech, Shanghai, China). Sections were immersed in hematoxylin (Servicebio, Wuhan, China), differentiated in 0.5% hydrochloric alcohol and mounted in neutral gum (Servicebio, Wuhan, China).

### Indirect immunofluorescence (IF)

Paraffin sections were dewaxed and then thermally repaired as previously described. After PBS washing, blocking was performed with BSA for 30 min. After incubation with primary antibodies overnight at 4 °C, the sections were incubated with secondary antibodies for 30 min at room temperature. Sections were incubated with DAPI at room temperature in the dark for 10 min, then sealed after treatment with fluorescence quencher for 5 min.

### Reverse transcription-polymerase chain reaction (RT-PCR)

RNA was extracted from tissue samples with RNA Isolater Total RNA Extraction Reagent (Vazyme, Nanjing, China). RT-PCR was performed by Applied Biosystems PCR (Thermo Scientific, Waltham, MA, USA) with One-Step RT-PCR Systems (Thermo Scientific, Waltham, MA, USA).

### Quantitative polymerase chain reaction (qPCR)

RNA was extracted from tissue samples with RNA Isolater Total RNA Extraction Reagent (Vazyme, Nanjing, China). A cDNA Synthesis Kit (Vazyme, Nanjing, China) was used to reverse-transcribe RNA into cDNA. qPCR was performed by Applied Biosystems (Thermo Scientific, Waltham, MA, USA) with ChamQ Universal SYBR qPCR Master Mix (Vazyme, Nanjing, China).

### Western blotting

The total tissue proteins were extracted with protein lysis buffer (Beyotime Biotechnology, Shanghai, China). The total protein concentration was determined with a BCA Protein Assay Kit (Beyotime Biotechnology, Shanghai, China). Protein samples were heat-denatured at 95 °C for 10 min. Proteins were separated by electrophoresis on 12% (or another percentage) SDS-polyacrylamide gels, then transferred to PVDF membranes. Subsequently, 5% non-fat milk powder (Bioprimacy, Wuhan, China) was used to block the membrane for 1 h. The membrane was incubated with primary antibodies overnight at 4 °C and then with secondary antibodies (Abclonal Technology, Wuhan, China) for 1 h at room temperature. A high sensitivity ECL chemiluminescence detection kit (Vazyme, Nanjing, China) was used for color development.

### ELISA

A Porcine chemokine ligand 5 Assay Kit (H496–1), Porcine chemokine ligand 2 Assay Kit (H318–1) and Macrophage inflammatory protein-1α Assay Kit (H110). were purchased from Nanjing Jiancheng Bioengineering Institute. Procedures were performed according to the manufacturer’s instructions.

### Transcriptomic analysis

With the assistance of Novogene, we performed the transcriptomic analysis of piglet thymus tissue based on Illumina platform. Total amounts and integrity of RNA were assessed using the RNA Nano 6000 Assay Kit of the Bioanalyzer 2100 system (Agilent Technologies, CA, USA). And mRNA was purified from total RNA by using poly-T oligo-attached magnetic beads. PCR amplification, the PCR product was purified, then the library was finally obtained. Ensure the quality of the library by using Agilent 2100 bioanalyzer. Clustering and sequencing by the Illumina NovaSeq 6000. Clean data (clean reads) were obtained by removing reads containing adapter, reads containing N base and low quality reads from raw data. Reads mapping to the reference genom by usingHisat2 (v2.0.5). The featureCounts v1.5.0-p3 was used to count the reads numbers mapped to each gene. Differential expression analysis of two groups was performed using the DESeq2 R package (1.20.0). Gene Ontology (GO) and KEGG pathways enrichment analysis of DEGs was implemented by the clusterProfiler R package (3.8.1). PPI analysis of DEGs was based on the STRING database.

### Statistical analysis

The immunohistochemical positive rate, TUNEL positive rate, thymic cortex area, thymic medulla area and thymic parenchyma area were calculated with Aperio ImageScope [v12.4.3.5008] (Leica Biosystems, Wetzlar, Germany). Statistical analysis was performed by paired two-tailed Student’s t test based on Graphpad Prism 9.0. The p value equal to lower than 0.05 was considered significant (*, *P* ≤ 0.05; **, *P* ≤ 0.01; ***, *P* ≤ 0.001; ****, *P* ≤ 0.0001).

### Drawing of figures

The figures in the discussion were drawn in FigDraw (www.figdraw.com).

## Results

### PRRSV-infected piglet model

**Fig. 1:**
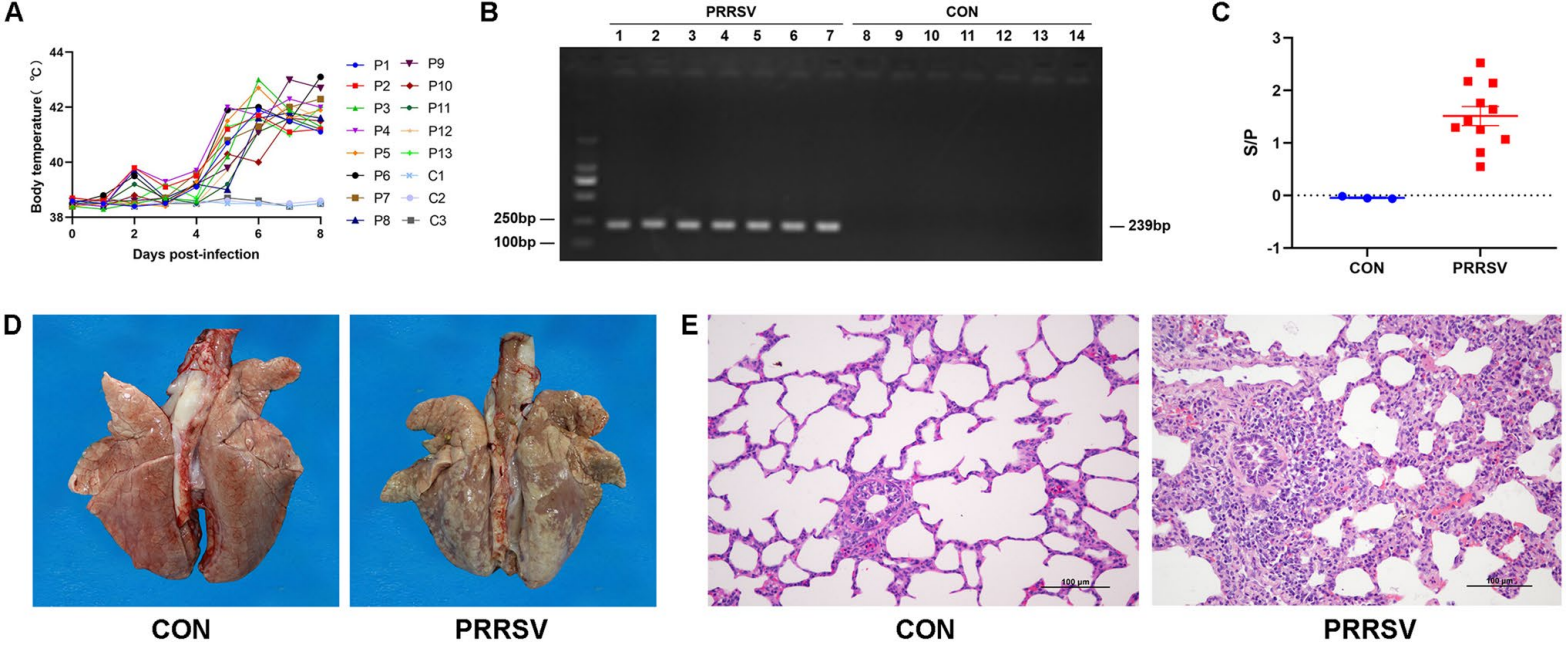
Establishment of a PRRSV-infected piglet model. **A** Body temperature of piglets. C1–C3: control group (*n* = 3). P1–P11: PRRSV-infected group (*n* = 11). **B** PRRSV nucleic acid test results. 1–7: PRRSV-infected group (*n* = 11). 8–14: control group (*n* = 3). 1,8: thymus; 2,9: hilar lymph node; 3,10: inguinal lymph node; 4,11: mandibular lymph node; 5,12: tonsil; 6,13: spleen; 7;14: lung. Each lane showed the detected result of mix samples of all same tissues in the group. **C** PRRSV antibody levels in the serum. CON: control group (*n* = 3). PRRSV: PRRSV-infected group (n = 11). **D** Macroscopic examination of lungs. The lungs in the control group were pink and soft. Irregular gray-white lesions were observed on the lung surfaces in the PRRSV-infected group. **E** Histopathological examination of lungs. Interstitial pneumonia was observed in the lungs in the PRRSV-infected group, with congestion and widening of lung stroma. H&E, 200 ×

All animals survived to the scheduled necropsies. The piglets in the control group had normal appetite, mental status and body temperature (approximately 38.5 °C) (Fig. 1A, C1-C3). The piglets in the PRRSV-infected group began to experience symptoms included depression, anorexia, high fever, sneezing and dyspnea at 3–4 days post-infection (dpi), and showed elevated body temperatures at 4 dpi, which reached 42–43 °C at 8 dpi (Fig. 1A, P1-P11).

The RT-PCR results indicated that PRRSV nucleic acids (239 bp) were present in the central immune organ of the thymus and in peripheral immune organs including hilar lymph nodes, inguinal lymph nodes, mandibular lymph nodes, the tonsils and the spleen (Fig. 1B). In evaluation of PRRSV serum antibody levels in piglets by indirect ELISA, the control group had S/P values less than 0 and were considered negative, whereas PRRSV-infected piglets had S/P values in the range of 1.0690 to 2.5249, thus meeting the condition for positivity of S/P ≥ 0.500 (Fig. 1C, Table 1).

**Table 1:** PRRSV antibody levels in serum

Sample	S/P	Result
C1	−0.0633	–
C2	−0.0532	–
C3	−0.0158	–
P1	1.0690	+
P2	2.1403	+
P3	1.4038	+
P4	1.2952	+
P5	1.7613	+
P6	2.1403	+
P7	1.2545	+
P8	2.1584	+
P9	1.6425	+
P10	2.5249	+
P11	2.1742	+

C1–C3: control group. P1–P11: PRRSV-infected group. “-” is negative; “+” is positive. S/P = (OD value of sample - average OD value of negative control)/(average OD value of positive control - average OD value of negative control)

Gross autopsy revealed irregular gray-white lesions on the lung surfaces in infected piglets (Fig. 1D). H&E-stained sections showed interstitial pneumonia in the infection group (Fig. 1E). On the basis of the clinical symptoms, detection of viral nucleic acids, serum antibody levels and pulmonary lesions in piglets, the PRRSV-infected piglet model was successfully constructed.

### Effects of PRRSV infection on immune organs in piglets

**Fig. 2:**
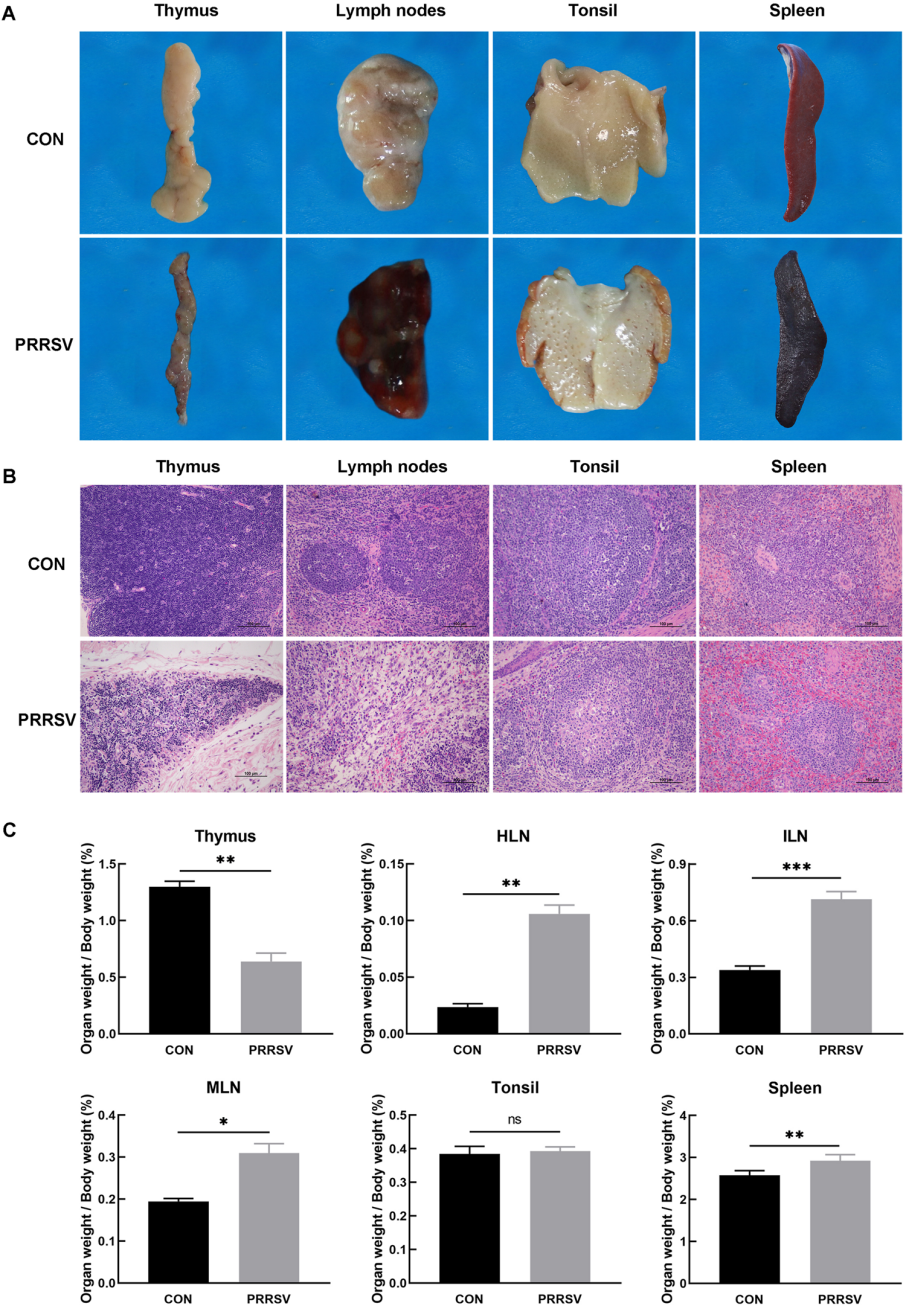
Effects of PRRSV infection on immune organs in piglets. Immune organ autopsy findings. (**A**) Macroscopic examination. The thymus glands in the PRRSV-infected group showed atrophy and scattered bleeding spots on the surfaces. The lymph nodes were swollen and bleeding. The tonsils were scattered with pinpoint-sized bleeding spots. The spleens showed swelling, and the surfaces were dark red. **B** Histopathological changes in immune organs (H&E, 200×). Massive necrotic thymocytes were observed in the thymus in the PRRSV-infected group. Lymphocytopenia was observed in lymph nodes with bleeding. Massive necrotic lymphocytes were observed in the centers of lymphoid nodules in the tonsils. Spleen red pulp congestion was observed. **C** Immune organ index. The organ index of the thymus was significantly lower, and the organ index of hilar lymph nodes (HLN), inguinal lymph nodes (ILN), mandibular lymph nodes (MLN) and spleen was significantly higher, in infected than control piglets. Organ index = organ weight/piglet body weight. CON: control group (n = 3). PRRSV: PRRSV-infected group (n = 11). ^*^P ≤ 0.05, ^**^P ≤ 0.01, ^***^P ≤ 0.001

Gross autopsy of PRRSV-infected piglets, compared with those in the control group, showed a significantly smaller thymus with scattered hemorrhagic spots on the surfaces. The lymph nodes of PRRSV-infected piglets were swollen and dark red; the tonsil surfaces were evenly scattered with pinpoint-sized hemorrhagic spots; and the spleens were enlarged and dark red (Fig. 2A).

Tissue sections were stained with H&E and observed through microscopy. Less cellularity and necrosis of lymphocytes were observed in the thymus in the infected piglets than the control group. In the infected group, the lymph nodes were hemorrhagic, structurally indistinct and lymphopenic; massive lymphocytes were necrotic in the center of the tonsillar lymphoid nodules; and splenic red pulp congestion was observed (Fig. 2B).

The organ index for immune organs (organ index = organ weight/piglet weight) indicated that the organ index of thymus (*P* ≤ 0.01) was significantly lower, and the organ index values of hilar lymph nodes (*P* ≤ 0.01), inguinal lymph nodes (*P ≤* 0.001), mandibular lymph nodes (*P* ≤ 0.05) and spleen (*P* ≤ 0.01) were significantly higher in infected than control piglets (Fig. 2C). The findings indicated that PRRSV infection severely affected the development of immune organs in piglets but had little effect on the tonsils.

Macroscopic findings, histopathological findings and organ index values indicated that PRRSV caused severe damage to the immune organs in piglets.

### Effects of PRRSV infection on the thymus in piglets

#### Thymus atrophy

**Fig. 3:**
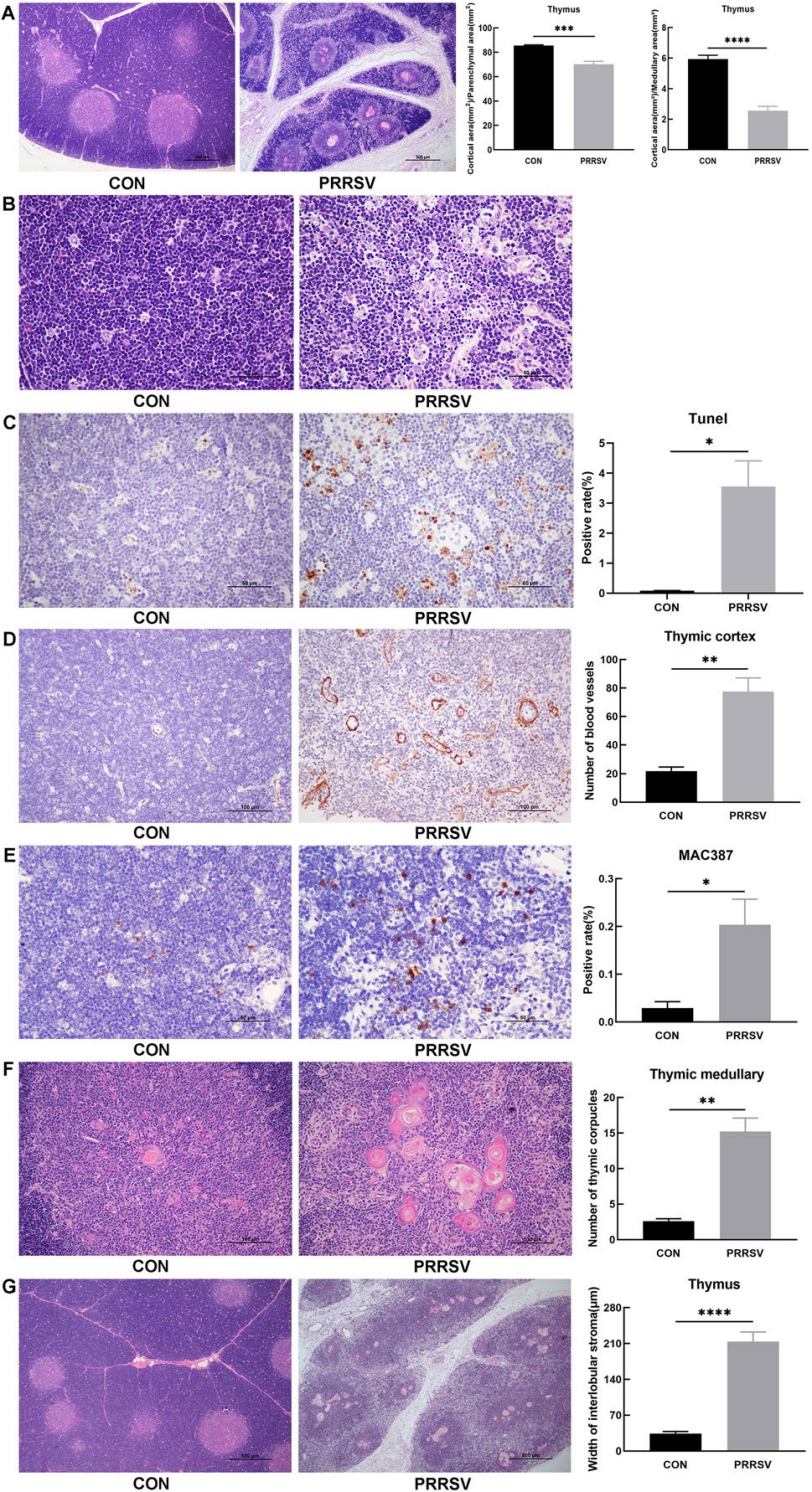
Effects of PRRSV infection on the thymus in piglets. **A** Thymus atrophy. Thymus paraffin sections showed that the thymic cortical area was significantly smaller in infected than control piglets, on the basis of H&E staining (H&E, 40×). The area ratio of the thymic cortex to thymic parenchyma and thymic cortex to thymic medulla in the infected group were significantly smaller than that in the control group. **B** Thymic cortical cell necrosis (H&E, 400×). Necrotic cells were observed in the thymic cortex, with clear nuclear pyknosis, in the PRRSV-infected group. **C** Apoptosis index, detected with TUNEL assays. The number of apoptotic cells in the thymic cortex in PRRSV-infected piglets was significantly greater than that in control piglets. **D** α-SMA IHC staining of the thymic cortex. α-SMA was used as an immunohistochemical blood vessel marker. The number of blood vessels in the thymic cortex in the PRRSV-infected group was significantly greater than that in control piglets. **E** MAC387 IHC staining of the thymic cortex. MAC387 was used as a macrophage immunohistochemical marker. The number of thymic cortical macrophages was significantly greater in PRRSV-infected than control piglets. **F** Number of thymic corpuscles in the thymic medulla. The number of thymic corpuscles in the thymic medulla in the PRRSV-infected group was significantly greater than that in the control group. **G** Interstitial width of thymic lobules. The interstitial width of thymic lobules in the PRRSV-infected group was significantly greater than that in the control group. CON: control group (*n* = 3). PRRSV: PRRSV-infected group (*n* = 11). ^*^P ≤ 0.05, ^**^P ≤ 0.01, ^***^P ≤ 0.001, ^****^P ≤ 0.0001

H&E staining indicated that the thymic cortical area was significantly smaller in the infected than the control piglets (Fig. 3A). Using the ImageScope measurement tool, we determined that the area ratio of thymic cortex to thymic parenchyma (*P* ≤ 0.001) and the area ratio of thymic cortex to thymic medulla (*P* ≤ 0.0001) in the infected group were significantly smaller than those in the control group (Fig. 3A). The results suggested that thymic atrophy occurred primarily in the cortical part of the thymus.

#### Thymic cortical cell necrosis and apoptosis

H&E staining revealed that the thymic cortex in the control group was structurally intact, whereas some cells in the thymic cortex in the infected group were necrotic with clear nuclear pyknosis or karyorrhexis (Fig. 3B). TUNEL assays demonstrated that the number of apoptotic cells in the thymic cortex in piglets infected with PRRSV was significantly greater than that in the control group (Fig. 3C, *P* ≤ 0.05). PRRSV led to necrosis and apoptosis of thymic cortical cells in piglets.

#### Elevated numbers of thymic cortical blood vessels

Smooth muscle actin (α-SMA) can be used as a vascular immunohistochemical marker. IHC staining was performed with antibody to α SMA, and the positive signals were brownish yellow. The number of thymic cortical vessels was greater in the PRRSV-infected group than the control group (Fig. 3D, *P* ≤ 0.01).

#### Elevated numbers of thymic cortical macrophages

MAC387 can be used as a macrophage immunohistochemical marker. IHC staining performed with an antibody to MAC387 showed a brownish yellow positive signal. The number of thymic cortical macrophages was significantly greater in PRRSV-infected than control piglets (Fig. 3E, *P* ≤ 0.05).

#### Thymic corpuscle hyperplasia of the thymic medulla

H&E staining and microscopic examination of thymus tissue sections showed a significantly greater number of thymic corpuscles in the thymic medulla in the infected than the control piglets (Fig. 3F, *P* ≤ 0.01).

#### Interstitial widening of thymic lobules

On the basis of the H&E staining results, microscopic observation revealed significantly wider interstitial thymus lobules in PRRSV-infected than control piglets (Fig. 3G, *P* ≤ 0.0001).

### Distribution of PRRSV in the thymus in infected piglets

**Fig. 4:**
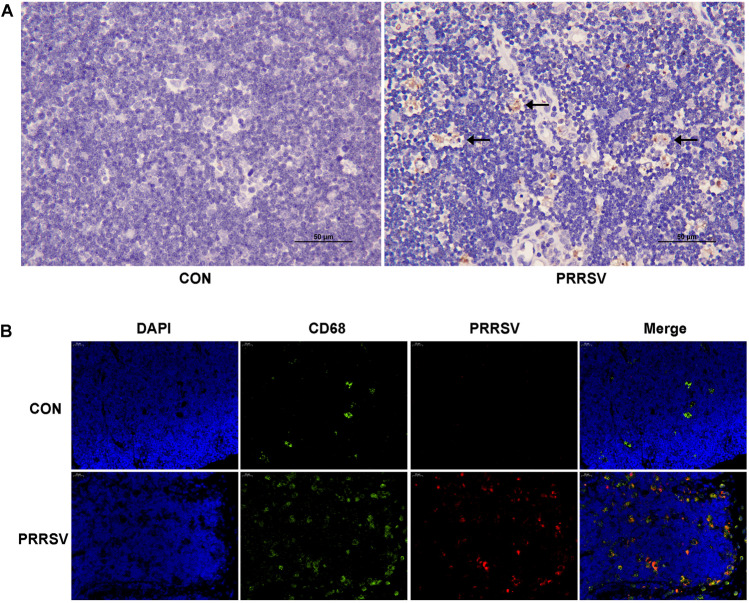
Distribution of PRRSV in the thymus. **A** Immunohistochemical staining of PRRSV (IHC, 400×). No positive signal was detected in the control group. PRRSV positive signals were apparent as a brownish-yellow color in the cytoplasm of thymic macrophages in the infected group (←). **B** Immunofluorescence. (IF, 200×). The nuclei emitted blue fluorescence, CD68 emitted green fluorescence, and PRRSV emitted red fluorescence. No PRRSV fluorescence signal was detected in the control group. PRRSV colocalization with macrophages in the infected group

The distribution of PRRSV in the thymus tissue was studied with IHC staining. No positive signal was detected in the control group, whereas PRRSV positive signals with a brownish-yellow color were observed in the infected group and were distributed in the cytoplasm of thymic macrophages (Fig. 4A). PRRSV was thus located in the cytoplasm of macrophages.

Indirect immunofluorescence was used to localize macrophages and PRRSV. No PRRSV fluorescence signal was detected in the control group, whereas PRRSV co-localized with macrophages in the infected group (Fig. 4B). This result again indicated that PRRSV infected thymic macrophages in piglets.

### Transcriptomic analysis

#### Differential expression analysis

**Fig. 5:**
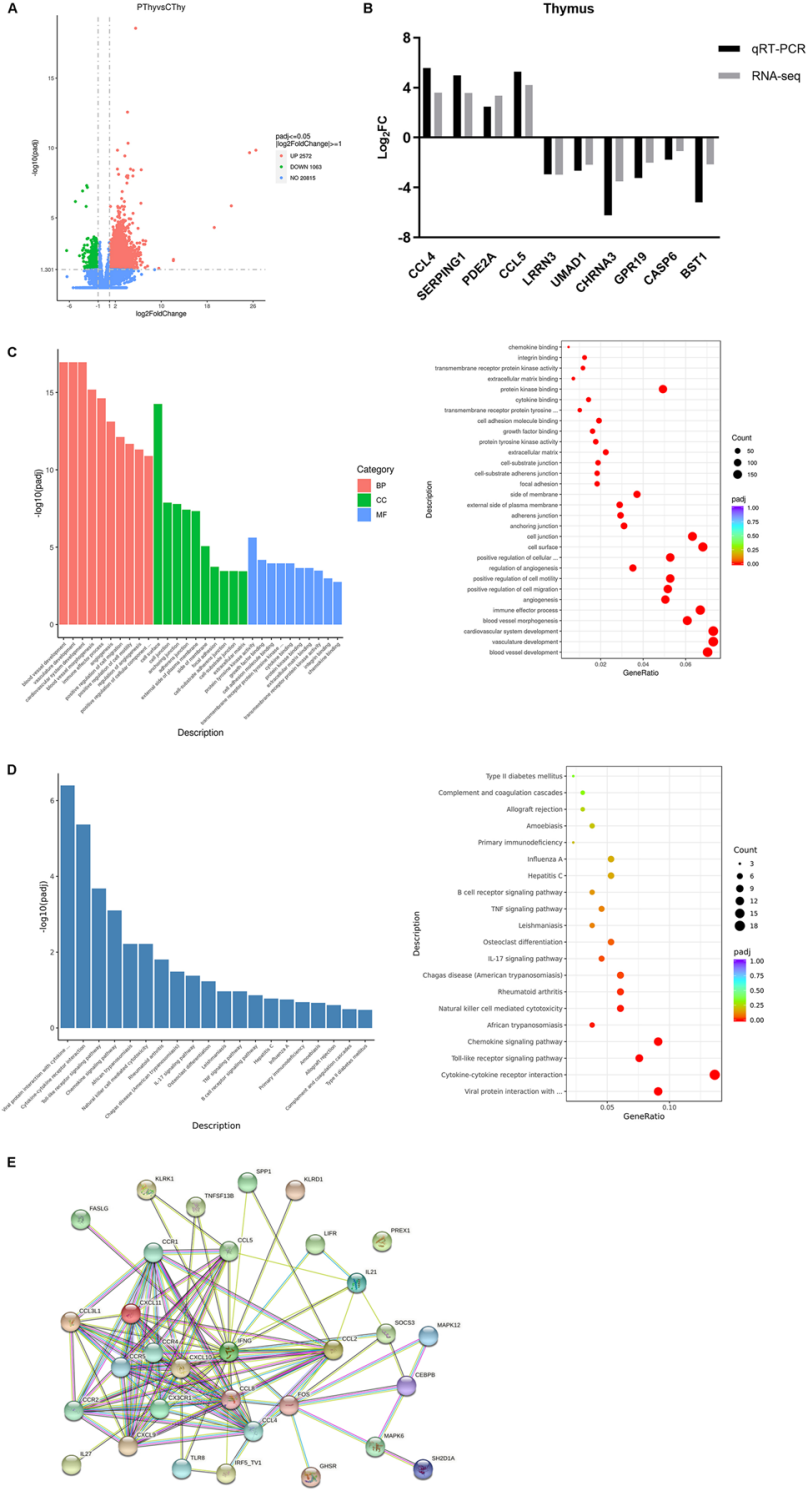
Transcriptomic analysis. **A** Volcano plot of differential expression analysis. The abscissa is the difference multiple, and the ordinate is the significance level. The dotted line represents the threshold line of DEG screening criteria. Green dots represent down-regulated genes, and red dots represent up-regulated genes. **B** Consistency between RNA-seq and qRT-PCR results. **C** GO enrichment analysis. Bar chart: the abscissa is the GO term, and the ordinate is the significance level of GO term enrichment. Different colors indicate different functional classifications. Bubble chart: the abscissa is the ratio of the number of DEGs mapping to the GO term to the total number of DEGs, and the ordinate is the GO term. The bubble size represents the number of genes mapping to the GO term. The color, from purple to red, represents a significant increase in enrichment. **D** KEGG enrichment analysis. Bar chart: the abscissa is the KEGG pathway, and the ordinate is the significance level of pathway enrichment. Bubble chart: the abscissa is the ratio of the number of DEGs mapping to the KEGG pathway to the total number of DEGs, and the ordinate is the KEGG pathway. The bubble size represents the number of genes mapping to the KEGG pathway. The color, from purple to red, represents a significant increase in enrichment. **E** Protein-protein interaction network. Nodes represent proteins, and lines represent the interactions between connected proteins

Differential expression analysis of two groups was performed with DESeq2 (1.20.0) with padj ≤0.05 and | log_2_ (fold change) | ≥ 1 as the threshold. A total of 3635 DEGs were screened, among which 2572 DEGs were up-regulated, and 1063 DEGs were down-regulated (Fig. 5A). Primers were designed for 10 randomly selected DEGs (Table 2), and the transcriptomic results were verified by qPCR. CCL4, SERPING1, PDE2A and CCL5 were significantly up-regulated, whereas LRRN3, UMAD1, CHRNA3, GPR19, CASP6 and BST1 were significantly down-regulated (Fig. 5B). These findings were consistent with the RNA-seq results (Fig. 5B), thus indicating that the sequencing results were credible.

**Table 2:** Primer sequences

Gene Name	Accession Number	Primer sequence (5′-3′)
TLR8	NM_214187.1	F: GTTTCGGATACCATTGCGGC R: AGCGACCGGTAGCCTTTTAC
IRF5	XM_021078963.1	F: CCCCCACATGGCACCCTATT R: GTTTGGCAGGACCTCAGAGAG
CCL2	NM_214214.1	F: ATCTTCAAGACCATCGCGGG R: TCAAGGCTTCGGAGTTTGGTT
CCL3L1	NM_001009579.1	F: CAGCTTCCTCGCAAATTCGT R: TCAGCTCCAGGTCAGAGATGT
CCL5	NM_001129946.1	F: AGGAAGCCTTGAGCCTGAAC R: AGGAGCCCTGGGAGGTTTTA
CCR1	NM_001001621.1	F: GCTCAGAAGCATGACCACCA R: ACCAAAGGTGATGGTCCGAG
CCR2	NM_001001619.1	F: CTACAGTGAGCCCTGCCAAA R: AGCATAGTGAGCCCAGAACG
CCR5	NM_001001618.1	F: TCTCGTTATACATGCAGCCCTC R: CCGAAGCAGGGTTTTGAGGA
CCL4	NM_213779.1	F: TTCACATACACCGTGCGGAA R: ACTCCTGGACCCAGTCATCA
SERPING1	NM_001123194.1	F: ACCACTCAACCCATCGAACC R: GCTGAATGACTCTCCTCGGG
PDE2A	XM_003482557.4	F: CAACTTTGCGGGGAGCTCTA R: CGTCCTGTCGTCAATGGGAA
LRRN3	XM_003134770.4	F: TGGCATCACCCCAACAGAAG R: CCGAATGGGCCTGAACATCT
UMAD1	XM_021102419.1	F: GTGACTGTAGCAAGCCCTGA R: CCAAGGTGAAGGGTACGTCG
CHRNA3	XM_001925760.6	F: AGTTGCTAGTCTCTCGCTGC R: TCTGCCCCATCATAGTCGGA
GPR19	XM_021092331.1	F: GCTGCCTTTTCATGTCGTCC R: GAGGCCGAGGAACTAAAGGA
CAS6	XM_013989349.2	F: AGCCTGGTTGGAAAACCCAA R: CACCTGGGTTAGGTTGGCAT
BST1	XM_003356861.5	F: CTCTCCAGGCACTCCATTCC R: ATAGAGAACGTCGCACAGGG
β-action	XM_021086047.1	F: GTGCGGGACATCAAGGAGAA R: CGTAGAGGTCCTTGCGGATG

#### GO enrichment analysis of differentially expressed genes

Gene Ontology (GO) enrichment analysis of DEGs was implemented with clusterProfiler (3.8.1). The biological processes were enriched mainly in blood vessel development, vasculature development, angiogenesis, immune effector process and positive regulation of cell migration. Cellular components were enriched mainly in cell surface; cell junction and anchoring junction. Molecular functions were enriched mainly in protein tyrosine kinase activity, growth factor binding, cytokine binding and chemokine binding (Fig. 5C).

#### KEGG enrichment analysis of differentially expressed genes

ClusterProfiler (3.8.1) was used to test the statistical enrichment of DEGs in KEGG pathways. Activated KEGG pathways were selected to construct bar and bubble graphs (Fig. 5D). DEGs were significantly enriched in pathways including the Toll-like receptor (TLR) signaling pathway, chemokine signaling pathway, IL-17 signaling pathway and TNF signaling pathway.

#### PPI analysis of differentially expressed genes

On the basis of the results of KEGG analysis, DEGs such as TLR8, CXCL9, IFNG, CCL2 and CCL5 in the significantly enriched TLR, chemokine signaling and TNF signaling pathways were selected for protein interaction analysis. The results (Fig. 5E) indicated that TLR8, IRF5, CCL2, CCL3 L1, CCL5, CCR2 and CCR5 interacted directly or indirectly, and are closely associated with the TLR pathway. On the basis of transcriptomic sequencing, the TLR pathway was significantly activated and thus might potentially play a role in the pathological changes in the thymus in PRRSV-infected piglets.

#### Effects of PRRSV on toll-like receptor pathways in the thymus in infected piglets

**Fig. 6:**
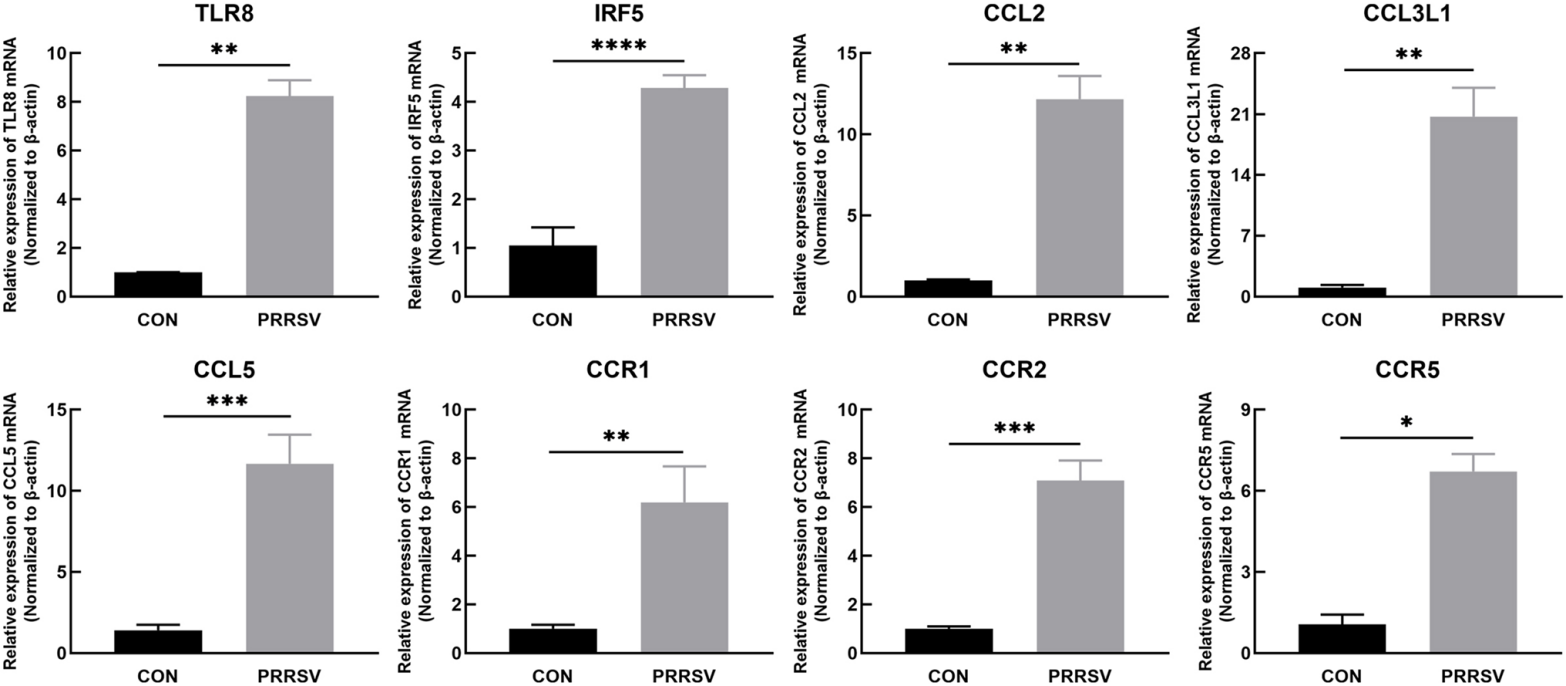
The mRNA expression of Toll-like receptor pathway related genes. The mRNA expression of TLR8, IRF5, CCL2, CCL3L1, CCL5, CCR1, CCR2, CCR5. CON: control group (*n* = 3). PRRSV: PRRSV-infected group (*n* = 11). ^*^P ≤ 0.05, ^**^P ≤ 0.01, ^***^P ≤ 0.001, ^****^P ≤ 0.0001

The mRNA expression in the thymus was detected by qRT-PCR. The mRNA expression levels of TLR8; IRF5; the chemokines CCL2, CCL3 L1 and CCL5; and their receptors CCR1, CCR2 and CCR5 was significantly greater in the infected group than the control group (Fig. 6). This result was consistent with the trends in the sequencing results.

**Fig. 7:**
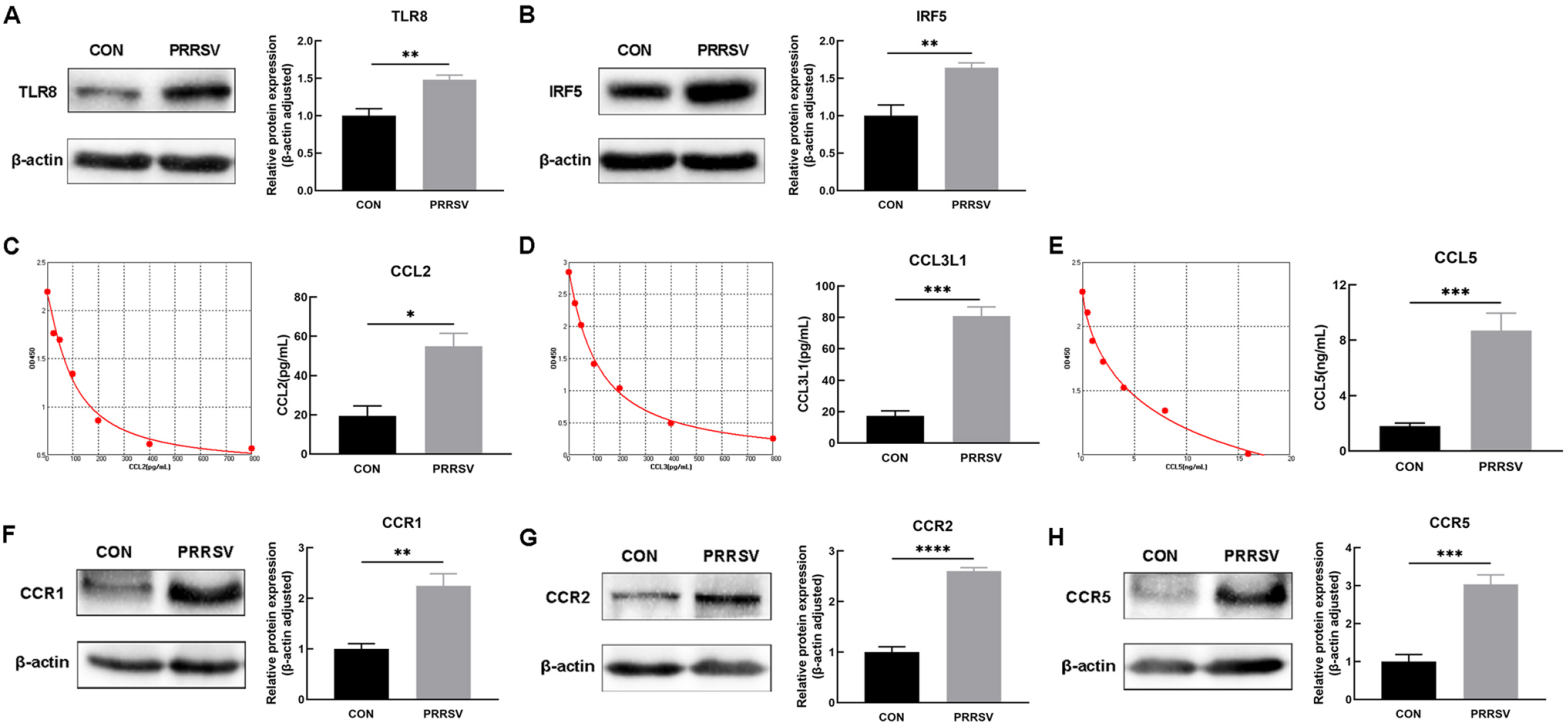
Protein expression of Toll-like receptor pathway related genes. **A**-**B** Protein expression of TLR8 and IRF5, determined with immunoblot assays. **C**-**E** Standard curve and protein expression of chemokines CCL2, CCL3L1 and CCL5, determined by ELISA. **F**-**H** Protein expression of chemokine receptors CCR1, CCR2 and CCR5, determined with immunoblot assays. CON: control group (*n* = 3). PRRSV: PRRSV-infected group (*n* = 11). ^*^P ≤ 0.05, ^**^P ≤ 0.01, ^***^P ≤ 0.001, ^****^P ≤ 0.0001. (*n* = 3)

The protein expression levels of DEGs in the TLR pathway were detected by western blotting. The protein expression levels of TLR8 (*P* ≤ 0.01) and IRF5 (*P* ≤ 0.01) in the thymus in the PRRSV infected group were significantly up-regulated (Fig. 7A-B). The expression levels of chemokines in the thymus were measured by ELISA, which revealed significant up-regulation of CCL2 (*P* ≤ 0.05), CCL3L1 (*P* ≤ 0.001) and CCL5 (*P* ≤ 0.001) expression (Fig. 7C-E). Western blot assays revealed significant up-regulation of protein expression of the chemokine receptors CCR1 (*P* ≤ 0.01), CCR2 (*P* ≤ 0.0001) and CCR5 (*P* ≤ 0.001) (Fig. 7F-H).

## Discussion

### Effects of PRRSV infection on immune organs in piglets

PRRSV infection of piglets causes primarily respiratory impairment and immunosuppression (Zhou and Yang 2010). PRRSV first infects porcine alveolar macrophages via the respiratory tract (Bordet et al. 2018; Ma et al. 2021), then invades host immune organs, mainly the thymus, spleen, lymph nodes and tonsils, via the blood and lymphatic circulation (Lunney 2016). In this study, macroscopic examination of PRRSV-infected piglets revealed a smaller thymus and a significantly lower thymus organ index than those in control piglets. Among the peripheral immune organs, the hilar lymph nodes, inguinal lymph nodes, mandibular lymph nodes and spleen were visually enlarged. Measurements indicated a significant increase in the organ index of these organs. PRRSV infection affected the development of immune organs in piglets but had no significant effect on tonsils.

Scattered bleeding spots were observed on the surfaces of the thymus and tonsils in infected piglets. The lymph nodes and spleen were enlarged, and the surfaces were dark red because of hemorrhage. Histological section observations indicated that the thymus in PRRSV-infected piglets had a diminished number of lymphocytes accompanied by cell necrosis. The lymph nodes were hemorrhagic, the cortical and medullary architecture was indistinct, and the number of lymphocytes was significantly diminished. Large numbers of necrotic lymphocytes in the center of the lymphoid nodule of the tonsil, and splenic red pulp congestion were observed. Macroscopic findings, organ indices and histopathological findings showed that PRRSV infection caused severe damage to the immune organs in piglets.

### Effects of PRRSV infection on the thymus in piglets

Studies have shown that PRRSV infection can lead to thymic atrophy (Wang et al. 2019), and the decrease in thymus volume can be as high as 90% (He et al. 2012). In this study, the area ratios of thymic cortex to thymic parenchyma, and the area ratios of thymic cortex to thymic medulla, were significantly smaller in the infected group than the control group, on the basis of by measurements and calculations. A large decrease in thymic cortical cells was observed by microscopic examination of H&E stained sections. The above results suggested that thymic atrophy occurred primarily in the cortical part of the thymus. TUNEL assay detection of apoptotic cells indicated that the number of apoptotic cells in the thymic cortex of PRRSV-infected piglets was significantly greater than that in control piglets, in agreement with findings reported by Ruedas Torres et al. (Ruedas-Torres et al. 2020). IHC staining revealed significant elevated number of blood vessels and macrophages in the thymic cortex in PRRSV-infected piglets.

IHC and IF indicated that PRRSV was located in the cytoplasm of macrophages. On the basis of these findings combined with the histopathological observation results, we speculated that PRRSV invaded the thymus in piglets and infected macrophages, thus leading to thymocyte apoptosis and necrosis. The animals initiated an immune defense, thus resulting in the proliferation of thymic cortical blood vessels. Monocytes in the blood migrated to the thymus and further developed into macrophages, thereby causing an increase in macrophages, that is, an increase in viral host cells. The virus further replicated and was then released and infected macrophages. This finding suggested that the pathological damage to the thymus caused by PRRSV resulted from the interaction between the virus and the animals’ immune system.

In the thymic medulla, thymic corpuscle hyperplasia was observed in infected piglets. Although thymic corpuscles are the characteristic structure of thymic medulla, their function has not been elucidated. However, thymi lacking thymic corpuscles cannot support T cell growth. Therefore, we hypothesized that viral infection led to a massive decrease in thymic lymphocytes, and, to compensate for the damage, thymic corpuscles proliferated, thereby promoting thymic lymphocyte development. In addition, H&E staining indicated that the interstitium of thymic lobules widened, which corresponded to thymic atrophy. The pathological changes finded in the thymus of piglets caused by PRRSV infection may be due to the direct effect of the virus or may be an indirect consequence intiated by the body’s immune response after infection.

### Transcriptomic analysis

Transcriptomic studies are often applied in the analysis of pathogenic mechanisms of pathogens (Joseph 2017; Chambers et al. 2018). Transcriptomics had been widely used to explore the molecular mechanisms of PRRSV pathogenicity. PRRSV-associated transcriptomic studies have often focused on lung or PAMs, whereas relevant transcriptomic analysis of thymic tissue has been less commonly reported (Cong et al. 2014; Li et al. 2015). In this study, we performed transcriptomic analysis on the thymus in control and PRRSV-infected piglets. Blood vessel development, angiogenesis and vasculature development were significantly enriched terms according to in GO enrichment analysis; these findings corresponded to the pathologic results indicating vascular proliferation in thymic cortex. In addition, the significant enrichment in immune effector processes and positive regulation of cell migration was also associated with the phenomenon and speculation of thymic cortical vascular proliferation and the increase in macrophages caused by the migration of monocytes from blood vessels to the thymic cortex.

KEGG enrichment analysis showed that DEGs were significantly enriched in the TLR signaling pathway, IL-17 signaling pathway and TNF signaling pathway. The results suggested that these signaling pathways may play roles in the pathogenic and immune evasion process of PRRSV as well as in host antiviral processes. TLRs activate innate and adaptive immune responses to viruses by releasing inflammatory cytokines and antiviral mediators (Kawai and Akira 2007). TLR8 recognizes viral single stranded RNA (Boisson and Casanova 2021). Studies have shown that PRRSV infection significantly increases TLR8 expression in the peripheral blood and brain medulla in hosts (Liu et al. 2009; Zhang et al. 2013). We found that PRRSV also led to significant up-regulation of TLR8 in the thymus, thus suggesting that TLR8 may mediate the interaction between virus and host cells in the thymus.

Transcription factors of the interferon regulatory factor (IRF) family mediate antiviral responses and play multiple functions in apoptosis, the cell cycle, tumorigenesis and gene regulation (Almuttaqi and Udalova 2019). In mammals, the IRFs family has nine members, IRF1–9 (Mamun et al. 2021). IRF5 is a signaling-dependent transcription factor that plays a key role in maintaining the inflammatory phenotype of macrophages (Albers et al. 2021). Corbin et al. (Corbin et al. 2020) have found that IRF5 induced the transformation of monocytes into macrophages in the intestine, and promotes the development of enterocolitis. In this study, the expression of IRF5 was significantly elevated in the thymus in PRRSV-infected piglets. Combined with the pathological results, these findings suggested that IRF5 might play a role in inducing the transformation of monocytes into macrophages.

Chemokines are involved in the immune and inflammatory responses, and play an important role in leukocyte migration under steady-state and inflammatory conditions (Sierra-Filardi et al. 2014). Monocyte chemotactic protein-1 (MCP-1/CCL2), macrophage inflammatory protein-1α (MIP-1α/CCL3L1) and chemokine ligand 5 (CCL5) belong to the monocyte/macrophage chemokine system, which mediates macrophage migration and infiltration (Zlotnik and Yoshie 2012). CCL2 binds CCR2 and mediates monocyte migration and macrophage recruitment (Sierra-Filardi et al. 2014). In addition to inducing macrophage transfer to inflammation sites, CCL3 plays a role in the thymus development and angiogenesis (Maurer and von Stebut 2004). CCL5 mediates the transfer of macrophages to inflammatory sites, and is an important ligand for CCR1 and CCR5. Pathways downstream of CCL5/CCR5, including NF-κB, HIF-α, RAS-ERK-MEK and TGF-β-Smad, are associated with biological processes such as apoptosis, cell metastasis, angiogenesis and inflammation (Marques et al. 2013). In this study, CCL2, CCL3L1, CCL5 and their receptors were significantly up-regulated in infected-piglets, and thus may play a role in thymocyte necrosis and apoptosis, vascular proliferation and macrophage proliferation.

**Fig. 8:**
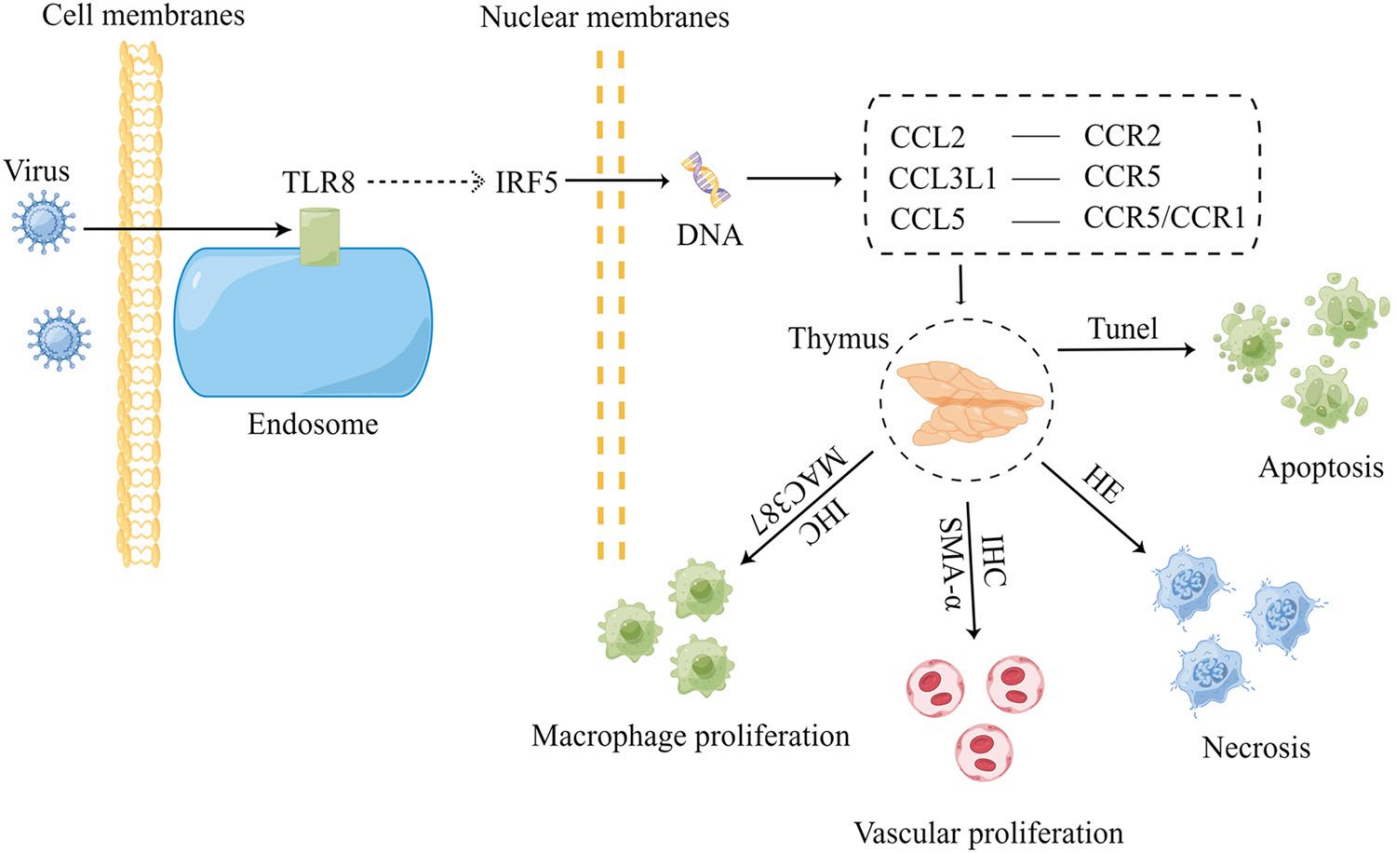
Roles of the Toll-like receptor signaling pathway in thymus injury caused by PRRSV infection

Previous studies showed that PRRSV infection result in thymic atrophy and thymocyte apoptosis (Ruedas-Torres et al. 2020; Wang et al. 2020). In this study, besides thymic atrophy and cell apoptosis, the histopathological findings included vascular and macrophage proliferation, thymic corpuscles hyperplasia and thymic lobules interstitum widened were also observed. Transcriptomic results showed that the expression of TLR8, IRF5, the chemokines CCL2, CCL3L1 and CCL5; and their receptors CCR1, CCR2 and CCR5 was significantly up-regulated in the thymus of PRRSV-infected piglets, thus suggesting that these cytokines were associated with the pathological processes of thymus injury. Previous studies showed that PRRSV infection significantly increases TLR8 expression in the peripheral blood and brain medulla in hosts (Liu et al. 2009; Zhang et al. 2013). In this study, the expression of TLR8 was significantly up-regulated in the thymus was detected. On the basis of these findings combined with the transcriptomic results, we conjectured that PRRSV invaded the thymus and caused up-regulation of TLR8 expression, which indirectly led to up-regulation of downstream IRF5 expression. IRF5 then entered the nucleus and affected gene expression, thus causing cells in the thymus to release cytokines such as CCL2, CCL3L1 and CCL5, which in turn induced the migration of monocytes from blood vessels to the thymic cortex and the development of macrophages. In addition, the interaction of chemokines with their receptors might have been involved in apoptosis, cell necrosis and vascular proliferation (Fig. 8). However, the exact mechanism of how PRRSV affects TLR8-mediated signaling pathway via IRF5 needs further study.

## Conclusions

PRRSV infection caused severe damage to the immune organs of piglets. Thymus injury was characterized by thymic cortical atrophy, cell apoptosis and necrosis, vascular hyperplasia, macrophage hyperplasia, thymic corpuscle hyperplasia, and interstitial widening of the thymic lobules. Transcriptomic results indicated the expression levels of TLR8, IRF5, CCL2, CCL3L1, CCL5, CCR1, CCR2 and CCR5 were significantly up-regulated in the thymus in PRRSV-infected piglets. These genes may be associated with pathological processes such as apoptosis and necrosis of thymic cortical cells, vascular proliferation and macrophage proliferation.

## Data Availability

The datasets that support the findings of this study have been deposited in NCBI with the accession number PRJNA919411.
